# Impaired Prefrontal-Amygdala Pathway, Self-Reported Emotion, and Erection in Psychogenic Erectile Dysfunction Patients With Normal Nocturnal Erection

**DOI:** 10.3389/fnhum.2018.00157

**Published:** 2018-04-24

**Authors:** Jianhuai Chen, Yun Chen, Qingqiang Gao, Guotao Chen, Yutian Dai, Zhijian Yao, Qing Lu

**Affiliations:** ^1^Department of Andrology, Nanjing Drum Tower Hospital, The Affiliated Hospital of Nanjing University Medical School, Nanjing, China; ^2^Department of Psychiatry, Nanjing Brain Hospital, The Affiliated Hospital of Nanjing Medical University, Nanjing, China; ^3^Key Laboratory of Child Development and Learning Science, Research Centre for Learning Science, Southeast University, Nanjing, China

**Keywords:** psychogenic erectile dysfunction, nocturnal penile tumescence, diffusion MRI, graph theory, prefrontal cortex, amygdala

## Abstract

**Background:** Neuroimaging studies have demonstrated that the prefrontal cortex and amygdala play an important role in sexual arousal (SA). However, little is known about the interactions between the prefrontal and cortex amygdala, which mediate the cognitive regulation of emotion and SA.

**Objective:** We seek to determine whether nocturnal erection of psychogenic erectile dysfunction (pED) patients are normal and whether there are changes of topological organization in the prefrontal-amygdala pathway of brain network in pED. In addition, whether there are correlations between network property changes and self-reported emotion and erection.

**Design, setting, and participants:** We used the RigiScan device to evaluate erectile function of patients and employed diffusion MRI and graph theory to construct brain networks of 21 pED patients and 24 healthy controls.

**Outcome measurements and statistical analysis:** We considered four nodal metrics and their asymmetry scores, and nocturnal penile tumescence (NPT) parameters, to evaluate the topological properties of brain networks of pED and their relationships with the impaired self-reported emotion and erection.

**Results and limitations:** All the pED patients showed normal nocturnal penile erection, however impaired self-reported erection and negative emotion. In addition, patients showed lower connectivity degree and strength in the left prefrontal-amygdala pathway. We also found that pED exhibited lower leftward asymmetry in the inferior frontal gyrus. Furthermore, patients showed more hub regions and fewer pivotal connections. Moreover, the degree of the left amygdala of pED showed significantly negative correlation with the self-reported erection and positive correlation with the self-reported negative emotion.

**Conclusions:** Together, these results suggest normal nocturnal erection in pED. However, abnormalities of brain network organization in pED, particularly in the left prefrontal-amygdala pathway, are associated with the impaired self-reported erection and negative emotion.

## Introduction

Psychogenic erectile dysfunction (pED) owing predominantly to psychological factors, has a significant effect on quality of life of both sufferers and their partners (Shamloul and Ghanem, 2013). Nocturnal penile tumescence (NPT) test has been considered as the gold standard for distinguishing psychogenic from organic ED (Elhanbly and Elkholy, 2012). However, the insomnia symptoms of some psychiatric diseases may cause false negativity of NPT test (Hirshkowitz and Schmidt, 2005). A more reliable and practical test is needed for the diagnosis of ED and for distinguishing psychogenic from organic ED. Moreover, normal erectile function is an integrated response under the control of the central and peripheral nervous system (Stoléru et al., 2012). Therefore, a better understanding of the underlying neuroanatomical correlates of pED may assist to identify biomarkers for clinical diagnosis for pED.

An emerging body of evidence has suggested that brain plays a central role in the control of male sexual arousal (SA), which is a multidimensional experience comprising both physiological and psychological processes (Stoléru et al., 2012). Previous studies investigating the neural correlates of man SA have revealed a broad network of brain regions that are activated or inactivated during the processing of visual erotic stimuli (Seo et al., 2010; Kim et al., 2013). The amygdala is essential for emotional processes (Madan et al., 2017); meanwhile, it is extensively interconnected with the prefrontal cortex (Gold et al., 2015), which is responsible for the cognitive aspects of emotional responses. In addition, prior model relates some brain regions to distinct components of SA, including cognitive, motivational, autonomic, and emotional components (Redouté et al., 2000; Ferretti et al., 2005; Cheng et al., 2015).

Neuroimaging studies have demonstrated that the amygdala and insula involve in SA (Kim et al., 2013). The involvement of amygdala is related to the specific hedonic quality of the emotional component of SA (Ferretti et al., 2005). Compared to viewing non-erotic emotional stimuli, bilateral amygdala are more activated during viewing erotic emotional stimuli (Karama et al., 2002). However, the regional cerebral blood flow of the right amygdala under the sexual stimulation of the penis is decreased when compared to that without penile erection (Georgiadis and Holstege, 2005). The activation of amygdala during SA and particularly during penile erection, is related to the appraisal process through which erotic stimulis are evaluated as sexual incentives (Karama et al., 2002). The level of SA elicited by the erotic stimuli is related to the extent of the attenuated vigilance and fear levels of sexually aroused men (Koukounas and McCabe, 2001). Since amygdala activation promotes vigilance and fear, deactivation of amygdala during sexual stimulation of the penis corresponds with an attenuated vigilance during SA (Georgiadis and Holstege, 2005). Therefore, amygdala deactivation may be crucial for SA to take place, while the active amygdala may preclude SA (Georgiadis and Holstege, 2005). And the amygdala is involved in the emotional component of SA model, especially the processes of the emotional aspects of penile sensations (Cheng et al., 2015). Previous neuroimaging study determines the presence of gray matter atrophy pattern in amygdala in pED patients (Cera et al., 2012).

Various lines of evidences have indicated that the medial and orbital parts of prefrontal cortex is also involved in man SA (Kim et al., 2013). The medial prefrontal cortex is related to subjective reports of self-relatedness during visual presentation of bodily erotic stimuli (Heinzel et al., 2006). The orbitofrontal cortex activation is correlated with both the cognitive and motivational components of the SA model (Cheng et al., 2015). In addition, the ventral medial, has been repeatedly reported to play a critical role in modulating negative mood states (Zald et al., 2002). Psychogenic ED patients show significantly decreased cortical thickness in widespread prefrontal regions, including the medial prefrontal, orbitofrontal, cingulate cortices (Cera et al., 2012). The activation of the prefrontal cortex may exert continuous inhibitory controls on SA, while the deactivation of these regions may release such inhibition during the development of SA (Poeppl et al., 2014). Moreover, the abnormal regions are found to be significantly correlated with male sexual functioning degradation, implying disassociations between the cognitive, motivational, and inhibitory networks of male SA in pED (Zhang et al., 2014). Especially, the medial prefrontal cortex, orbitofrontal, and cingulate cortex which are known to mediate the inhibitory control of male SA, show abnormally increased activations during sexual stimuli in pED patients (Kim et al., 2013).

However, alterations in the topological organization of white matter structural brain networks in pED patients are still unclear (Zhao et al., 2015). In this study, we seek to determine whether there are changes of topological organization in the prefrontal-amygdala pathway of brain network in pED patients and to clarify the relationship between the nocturnal erections and the altered brain regions. Finally, correlations between network property changes and self-reported emotion and erection are explored.

## Materials and methods

### Subjects

Patients who visited the inpatient's clinic for sexual dysfunctions of the department of andrology, Nanjing Drum Tower Hospital were recruited for this study. The sample consisted of 21 pED patients and 24 healthy controls (HC). All subjects were right handed and the age ranged from 20 to 45 years. All patients were in a stable heterosexual relationship for at least 1 year.

Patients were enrolled according to the following criterias: (1) normal morning and nocturnal penile erection rated by RigiScan test; (2) normal penile hemodynamics rated by the duplex doppler ultrasonography with peak systolic velocity (PSV) before and after intracavernous injection (ICI) of prostaglandin (Alprostadil) (However, so far, there was no article that analyzed the MRI data by coupling to induced sexual response by intracavernous injection. And we would explore this problem in our future research). Moreover, all patients were enrolled after (1) a detailed history taking (including medical, sexual, and relevant drug history); (2) basic laboratory tests; (3) the State-Trait Anxiety Inventory (STAI) were implemented to evaluate psychophysical status including the level of the self-reported positive and negative emotion; (4) sexual functioning assessed by the International Index of Erectile Function (IIEF)(Rosen et al., 1997).

Exclusion criterias were as follows: (1) being diagnosed as organic ED; (2) serious psychiatric, neurological, cardiovascular, respiratory, gastrointestinal, or renal diseases; (3) drug or alcohol dependence; (3) use of any medication that might affect sexual function during the previous 30 days; (4) any contraindications to undergo MRI scan.

This study was approved by the ethics committee of Nanjing Drum Tower Hospital (Program number (81571430): Version 1). Written informed consents were obtained from all subjects.

### Erectile function assessment

#### Rigiscan analysis

In all patients, NPT was performed during two nights using RigiScan device. The loops of the RigiScan device which could contract pressure to the penis and allow the measurement of rigidity, were applied to the tip and base of the penis. So RigiScan device could provide the penile tumescence and rigidity of both penile tip and base activity during sleep. The best result of two NPT tests was used for the statistical analysis. The erectile activity was recognized as an erectile event at least a 20% increase in the base loop circumference or at least a 60% increase in the tip penile rigidity and lasting for at least 3 min in duration. The leve of erectile activity was measured by the following parameters: number of erectile episodes, duration of erectile episodes (min), duration of rigidity > 60%, tumescence (TAU), and rigidity (RAU) activity units. The measurements of RAU (the product of elapsed time multiplied by the associated rigidity, ranging from 0 to 120) and TAU (penile circumference: the increase in tumescence over baseline tumescence, ranging from 0 to 120) could facilitate the interpretation of the time-dependent nature of rigidity and tumescence.

#### IIEF-5

We also selected IIEF-5 to assess the erectile function of all the ED patients. The IIEF-5 was a widely used self-report measure for the evaluation of male erectile function, which had been demonstrated to have high sensitivity and recommended as a primary test for evaluating the ED severity.

### Image acquisition

The MRI dataset were acquired using 3.0 T Siemens Trio scanner (Siemens AG, Erlangen, Germany). T1-weighted images were acquired with the following parameters: repetition time (TR) = 1,900 ms, echo time (TE) = 2.48 ms, slice thickness = 1 mm, number of slices = 176. The parameters of DTI images were as follows: TR/TE = 6,600 ms/93 ms, thickness/gap=3 mm/3 mm. Diffusion-weighted images were acquired along 30 non-collinear directions with b value = 1,000 s/mm^2^ and an additional image without diffusion weighting (b = 0 s/mm^2^) was also measured. The observers and the workers of magnetic resonance scanner were blinded.

### Image preprocessing

The MRI images were processed with the diffusion toolbox of the Functional MRI of the Brain (FMRIB) Software Library. Before data processing, eddy current distortions and motion artifact were corrected. The main pipeline processing steps included diffusion tensor estimation, FA calculation and fiber tract reconstruction (Chen et al., 2016). Diffusion toolkit toolbox (http://www.trackvis.org) was implemented to reconstruct the fiber tracts. The whole-brain fiber tracking was performed based on the Fiber Assignment by Continuous Tracking (FACT) algorithm and the propagation was terminated if a minimum tracking turning angle was larger than50° or a voxel with FA below 0.2 was encountered (Qin et al., 2014).

### Network construction

The Automated Anatomical Labeling (AAL) template with 90 regions was employed to define the nodes of the network (Tzourio-Mazoyer et al., 2002). The interregional fibers between all possible pairs of brain regions were defined as the edges of the network. Moreover, we set a threshold value for defining the edges of the network: at least two fibers passing through their respective regions with the fiber length greater than 5 mm were found (Chen et al., 2016). After that, the undirected binary and FA weighted anatomical brain networks were constructed and for further network analyses (Qin et al., 2014).

### Network analysis

#### Regional nodal parameters

Firstly, the network analysis had been validated previously, see our previous references (Qin et al., 2014; Chen et al., 2016). To examine the regional characteristics of brain networks, we considered four nodal metrics: the nodal degree *D(i)*, the nodal connectivity strength *S(i)*, the nodal local efficiency *Eloc(i)*, and the noal global efficiency *Eglo(i)* (Rubinov and Sporns, 2010). *D(i)* and *S(i)* reflected how strong node *i* was connected to the rest of the network. *Eloc(i)* reflected how well the information was transferred within the neighbors of node *i*. Higher value of *Eglo(i)* suggested more efficient for information transfer through the whole brain.

#### Asymmetry score

In order to characterize asymmetry for the noal measures [*D(i), S(i), Eglo(i)*, and *Eloc(i)*], the asymmetry of all nodal parameters were evaluated by the following asymmetry score:

$$\text{A}=100\times \frac{\text{X}\left(\text{L}\right)-\text{X}(\text{R})}{0.5\times (\text{X}\left(\text{L}\right)+\text{X}(\text{R}))}$$

where X(L) and X(R) were the parameters of the node *i* in the left and right hemispheres, respectively (Tian et al., 2011). Positive asymmetry scores indicated leftward asymmetry (X(*i*) was more prominent over the left hemisphere) and vice versa. Asymmetry score allowed us to look at differences between the left and right hemispheres.

#### Hub classification

*D(i)* and participation index *P(i)* were proposed to determine the role of a node in the network. *P(i)* expressed its distribution of intra- vs. extra-modular connections (Sporns et al., 2007). We classified nodes with *D(i)*≥*D*(mean)+*D*(SD) as network hubs and nodes with *D(i)*<*D*(mean)+*D*(SD) as non-hubs (Rosen et al., 1997). Hub nodes were then more finely characterized by using the values of *P(i)*. Considering only high-degree nodes, we classified nodes with *P(i)* ≥ 0.3 as connector hubs and nodes with *P(i)* < 0.3 as provincial hubs (Sporns et al., 2007).

#### Edge betweenness centrality

Edge betweenness centrality (*E*_*bc*_) identified critical paths in efficient communication of the brain network. *E*_*bc*_ expressed how central the edge was located in the network. Network edge with high *E*_*bc*_ were considered pivotal edges in the network and occupied a central positions in the network. We classified edges with *E*_*bc*_≥*E*_*bc*_(mean)+ *E*_*bc*_ (SD) as critical paths of the network (Chen et al., 2008).

### Statistical analysis

ANCOVA with the negative emotion score of STAI as covariant was performed to determine whether there were significant differences in any of the four nodal metrics and their asymmetry scores between groups. To address the problem of multiple comparisons in the nodal metrics, a false discovery rate (FDR, *q* of 0.05) multiple comparisons correction was performed. Furthermore, a one-sample *t*-test was performed to determine whether the asymmetry scores of the four nodal metrics within group were significantly different from zero. In addition, the relationships between the altered metrics and the self-reported emotion and erection of pED were explored by the *Pearson* correlation analysis. A significance threshold of *P* < 0.05 (uncorrected) was used for testing the brain network metrics. Moreover, compared with previous fMRI studies, there were enough patients/controls included in our study (Zhang et al., 2014; Zhao et al., 2015).

## Results

### Demographic and clinical data

The demographic, psychiatric characteristics of the subjects were showed in Table 1.

**Table 1:** Demographic and clinical characteristics of pED and HC.

**Variables**	**pED**	**HC**	***P*-value**
Sample size	21	24	–
Age (years)	29.38 ± 5.11	31.92 ± 6.45	0.16
Education (years)*	13.52 ± 1.83	14.29 ± 1.57	0.24
Score of IIEF	9.71 ± 5.71	23.92 ± 1.02	< 0.05
Negative emotion score of STAI	42.29 ± 11.54	35.96 ± 7.82	0.04
Positive emotion score of STAI	44.19 ± 15.10	44.38 ± 13.78	0.97

Data are presented as the range of mean ± SD (standard deviation). pED, the psychogenic erectile dysfunction patients group; HC, the healthy controls group; IIEF, International Index of Erectile Function; STAI, State-Trait Anxiety Inventory. The P values were obtained by two-sample two-tailed t test or Mann-Whitney U*. The P value more than 0.05 indicated no statistically significant difference between the two groups.

### NPT parameters of pED

**Figure 1:**
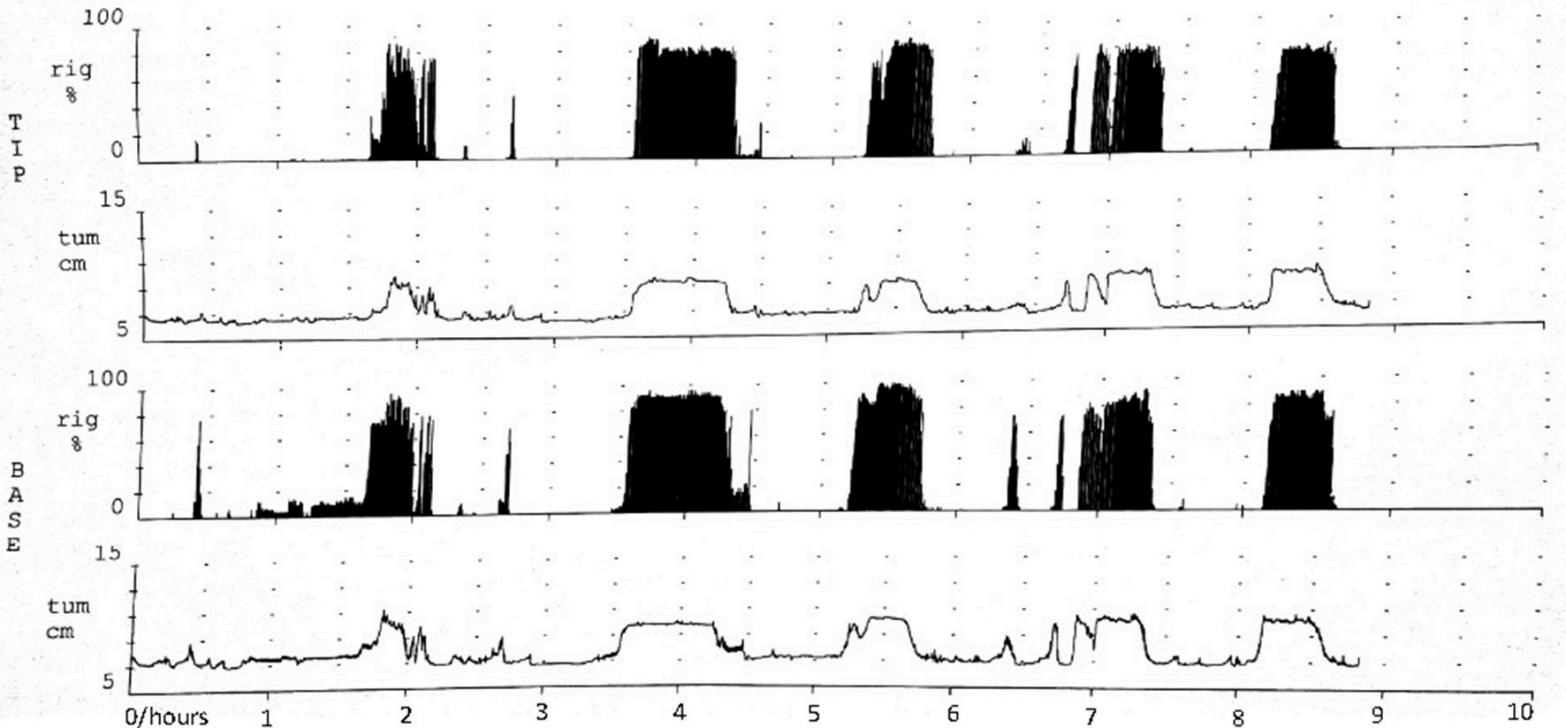
Normal morning and nocturnal penile erection for one pED patient rated by RigiScan test. TIP and BASE: the tip and base of penile. rig and tum: the rigidity and tumescence of penile. Abscissa represented time; Ordinate represented the tumescence or rigidity of penile.

The NPT parameters of pED were showed in Table 2. The parameters,including number of erectile episodes, duration of erectile episodes (min), duration of rigidity > 60%, TAU, and RAU were measured. All the pED patients showed normal morning and nocturnal penile erection rated by RigiScan test (showed in Figure 1).

**Table 2:** The NPT parameters of pED.

**Variables**	**pED**
**RIGISCAN TEST**	
Number of erection events	5.90 ± 1.95
Duration of erectile episodes (min)	112.38 ± 53.22
Duration of rigidity > 60%	89.52 ± 39.14
**TIP**	
Average rigidity	75.67 ± 11.68
RAU	79.57 ± 31.61
TAU	42.62 ± 21.79
**BASE**	
Average rigidity	61.81 ± 11.07
RAU	68.52 ± 34.27
TAU	41.24 ± 23.60

*Data are presented as the range of mean ± SD (standard deviation). pED, the psychogenic erectile dysfunction patients group; RAU, rigidity activity unit; TAU, tumescence activity unit*.

### Node-level properties analysis

#### Degree *D(i)*

**Figure 2:**
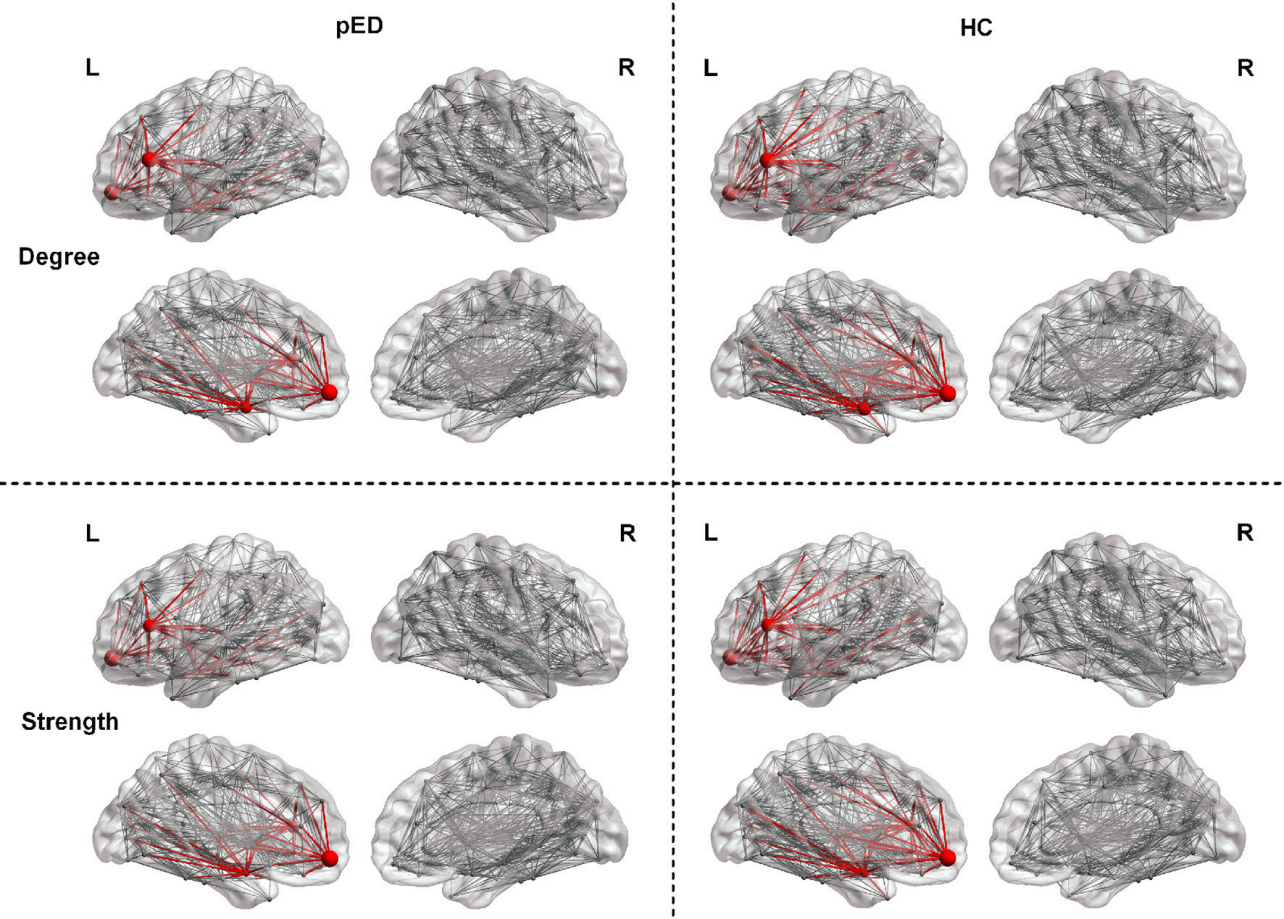
Regions exhibite significant differences in the regional nodal parameters, including *D(i)* and *S(i)*. L, left; R, right. The node size indicats the nodal degree and strength. The red nodes represente regions that showed significant differences after FDR correction. The red lines represente connections that connected with the above regions.

We found that pED showed decreased degree predominantly located in the left frontal lobe regions including the inferior frontal gyrus, triangular part [IFGtriang], superior frontal gyrus, medial orbital [ORBsupmed], and amygdala [AMYG]. All of these regions were the components of the left prefrontal-amygdala pathway. And all the above effects survived FDR correction (Figure 2).

#### Strength *S(i)*

Results of nodal strength analyses showed that pED exhibited significantly lower connectivity strength in the left frontal lobe regions (IFGtriang and ORBsupmed) and subcortical region (AMYG) (FDR corrected). All of these regions were the components of the left prefrontal-amygdala pathway (Figure 2).

#### Local efficiency *Eloc(i)*

In addition, pED did not show strong alterations in nodal local efficiency. Group comparison revealed increased nodal local efficiency of pED in three brain regions, including the right frontal lobe regions(the superior frontal gyrus, dorsolateral [SFGdor], and supplementary motor area [SMA]) and occipital lobe region (the calcarine fissure [CAL]). Decreased nodal local efficiency in pED were predominantly located in two regions of the right temporal lobe regions (the heschl gyrus [HES] and inferior temporal gyrus [ITG]). However, none of these showed significant differences after FDR correction. Not finding strong effects on nodal local efficiency suggested that local connectivity and efficiency were relatively intact in pED.

#### Global efficiency *Eglob(i)*

Additional statistical analysis revealed an abnormal trend in nodal global efficiency of pED, predominately in the frontal lobe regions(increased nodal global efficiency: the right superior frontal gyrus, medial [SFGmed], ORBsupmed; decreased nodal global efficiency: the left IFGtriang). However, effects also did not survive FDR correction.

### Asymmetry of node-level properties

In order to characterize the hemispheric asymmetry, we evaluated the asymmetry scores of four nodal parameters, including *D(i), S(i), Eloc(i)*, and *Eglob(i)*.

#### The asymmetry score of *D(i)*

**Figure 3:**
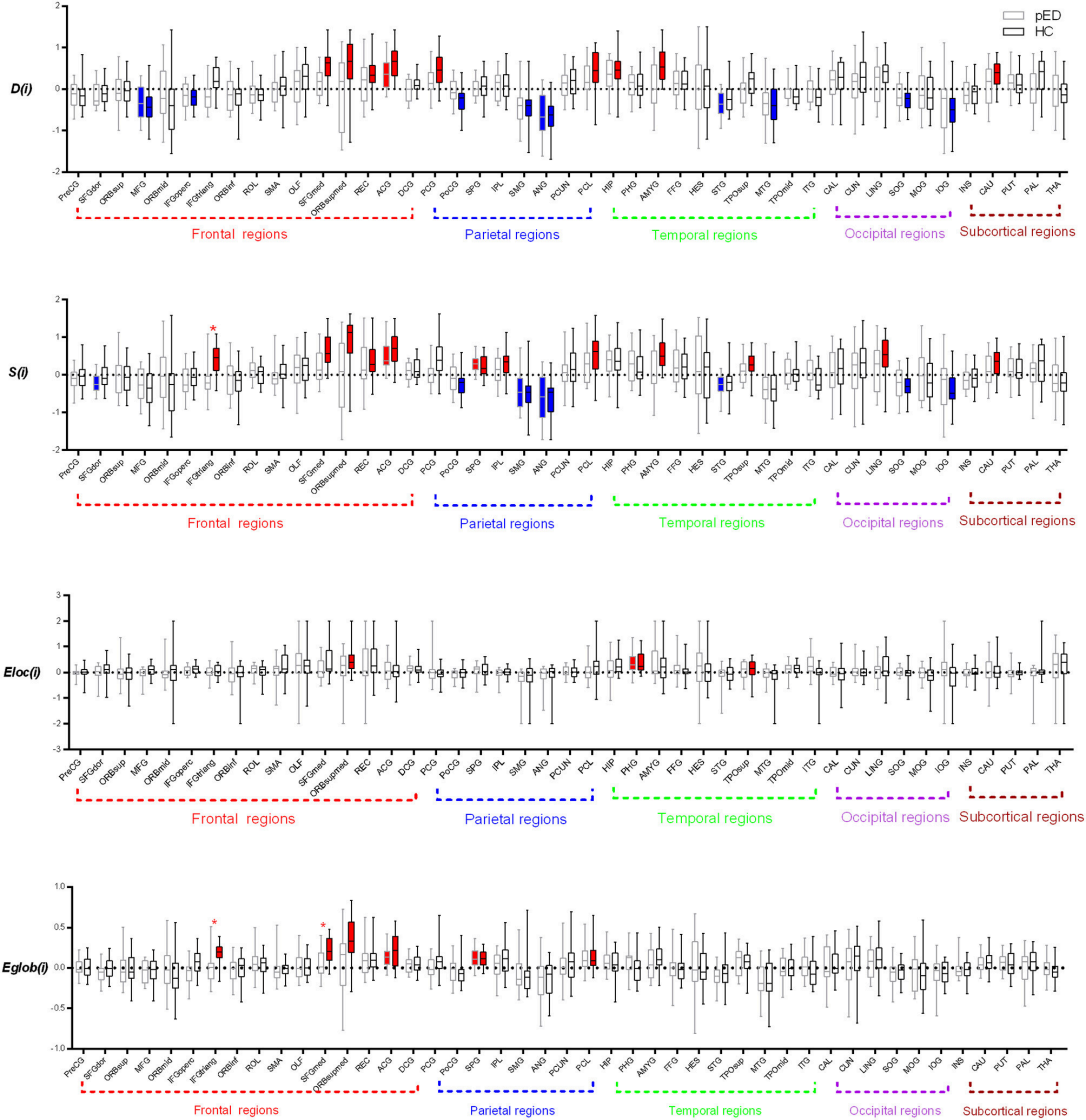
Regions exhibite significant hemispheric asymmetry differences of the regional nodal parameters, including *D(i), S(i), Eloc(i)*, and *Eglob(i)*. The red color represents significant leftward asymmetries, and the blue color represents significant rightward asymmetries. The dashed line indicats the threshold (mean+*SD*) of asymmetries. ^*^Represents FDR corrected regions.

In the pED group, we only found that 1 brain region exhibited significant leftward asymmetries of *D(i)*: the frontal lobe region (the anterior cingulate gyrus [ACG]). Regions with significant rightward asymmetries of *D(i)* were predominantly located at the frontal lobe region (the middle frontal gyrus [MFG]), parietal lobe region (the angular gyrus [ANG]), and temporal lobe region (the superior temporal [STG]) (Figure 3).

We found that nine brain regions exhibited significant leftward asymmetries of *D(i)* in HC group, mainly involving the frontal lobe regions (SFGmed, ORBsupmed, the gyrus rectus [REC], and ACG), parietal lobe regions (the posterior cingulate gyrus [PCG] and paracentral lobule [PCL]), temporal lobe regions (the hippocampus [HIP] and AMYG), and subcortical regions (caudate nucleus [CAU]). Regions with significant rightward asymmetries of *D(i)* were predominantly located at the frontal lobe regions (MFG and the inferior frontal gyrus, opercular part [IFGoperc]), parietal lobe regions (the postcentral gyrus [PoCG], SMG, and ANG), temporal lobe region (the middle temporal gyrus [MTG]), and occipital lobe regions (the superior occipital gyrus [SOG] and inferior occipital gyrus [IOG]) (Figure 3).

When comparing the asymmetry score of *D(i)* between pED and HC, we observed no significant group-related difference of the asymmetry score of *S(i)* (Figure 3).

#### The asymmetry score of *S(i)*

In the pED group, we found that two brain regions exhibited significant leftward asymmetries of *S(i)* mainly involving the frontal lobe region (ACG) and the parietal lobe region (the superior parietal gyrus [SPG]). Regions with significant rightward asymmetries of *S(i)* were predominantly located at the frontal lobe region (the superior frontal gyrus, dorsolateral [SFGdor]), the parietal lobe regions (the supramarginal gyrus [SMG] and ANG), and the temporal lobe region (STG) (Figure 3; more information see Supplementary Files).

We found that 12 brain regions exhibited significant leftward asymmetries of *S(i)* in the HC group, mainly involving the frontal lobe regions (IFGtriang, SFGmed, ORBsupmed, REC, and ANG), the parietal lobe regions (SPG, the inferior parietal gyrus [IPL] and PCL), the occipital lobe region (the lingual gyrus [LING]), the temporal lobe regions (the temporal pole: superior temporal [TPOsup] and AMYG), and the subcortical region (CAU). Regions with significant rightward asymmetries of *S(i)* were predominantly located at the parietal lobe regions (PoCG, SMG, and ANG) and the occipital lobe regions (SOG and IOG) (Figure 3; more information see Supplementary Files).

When comparing the asymmetry score of *S(i)* between pED and HC, we observed a significant group-related difference only in the frontal lobe region (IFGtriang) (FDR corrected) (Figure 3). IFGtriang of HC tended to exhibit stronger connectivity in their left hemispheres.

#### The asymmetry score of *Eloc(i)*

Significant leftward asymmetry score of *Eloc(i)* was observed only in the temporal lobe region (the parahippocampal gyrus [PHG]) in the pED group. Three brain regions of HC had been observed to be leftward asymmetric [*Eloc(i)*], involving the frontal lobe region (ORBsupmed) and the temporal lobe regions (PHG and TPOsup). However, no significant rightward asymmetry of *Eloc(i)* was observed in either group. Moreover, no significant group-related difference of the asymmetry score of *Eloc(i)* was found (Figure 3).

#### The asymmetry score of *Eglo(i)*

We observed that two brain regions (ACG and SPG) exhibited significant leftward asymmetries of *Eglo(i)* in the pED group. Six brain regions of HC had been observed to be leftward asymmetric [*Eglo(i)*], involving the frontal lobe regions (IFGtriang, SFGmed, ORBsupmed, and ACG) and the parietal lobe regions (SPG and PCL). However, no significant rightward asymmetry of *Eglo (i)* was observed in either group. We observed significant group-related difference of the asymmetry score of *Eglo(i)* in the frontal lobe region (IFGtriang) (FDR corrected). IFGtriang of HC tended to be more globally efficient in their left hemispheres (Figure 3).

### Hub classification

**Figure 4:**
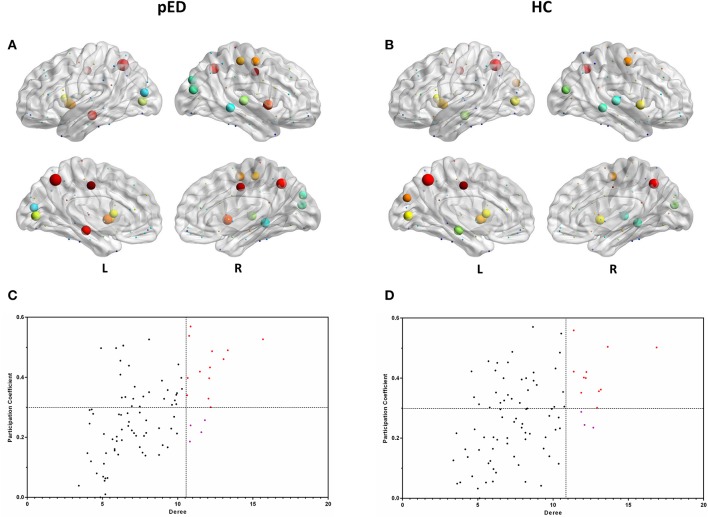
Hubs regions determined by degree and participation index. **(A,B)** show the hubs in the brain networks of pED and HC. Node size represents the magnitude of their degree *D(i)* and node color represents the magnitude of their participation index *P(i)*. **(C,D)** show the values of their *D(i)* and *P(i)*. The correlations were based on measurements in 21 patients and some of the points were superimposed.

In both groups, the majority of connector hubs were consistently found in the left insula [INS], median cingulate gyri [DCG], HIP, calcarine fissure cortex [CAL], precuneus [PCUN], CAU, putamen [PUT] and the right precental gyrus [PreCG], INS and PCUN, while only the right middle occipital gyrus [MOG], and MTG were classified as provincial hubs (Figure 4).

Three right brain regions including DCG, PoCG, and STG were identified as the connector hubs, and the right SOG, the left MOG were classified as provincial hubs only in pED. Additionally, we also noted that the left cuneus [CUN] was identified as the connector hubs only in HC, and the right STG was classified as provincial hub in HC (Figure 4).

### Edges betweenness centrality

**Figure 5:**
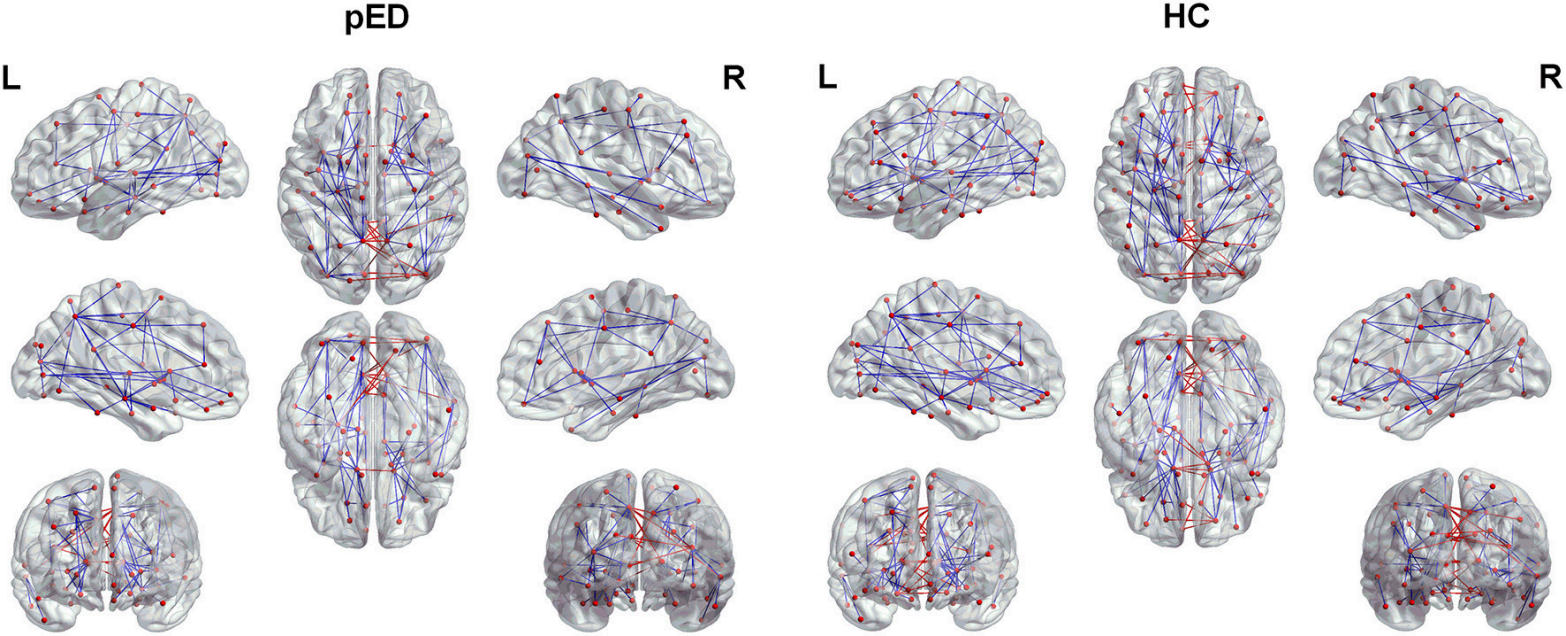
The important connections (*E*_*bc*_≥*E*_*bc*_(mean)+*E*_*bc*_ (SD)) for pED and HC. The blue lines represent intra-hemispheric connections, and the red lines represent inter-hemispheric connections.

The top 15 ranked connections for pED and HC were demonstrated in Table 3. Futhermore, edges were identified as the bridges in the network if their *E*_*bc*_ values were at least one standard deviation (*SD*) greater than the average *E*_*bc*_ of the network (*E*_*bc*_>mean + *SD*). The important connections (*E*_*bc*_≥*E*_*bc*_(mean)+*E*_*bc*_ (SD)) for pED and HC were shown in Figure 5. One hundred connections were identified as bridges in pED, including 13 inter-hemispheric and 87 intra-hemispheric connections; while 126 connections were identified as bridges in HC, including 24 inter-hemispheric and 102 intra-hemispheric connections. Moreover, 36 connections were identified as bridges in the right hemisphere and 51 in the left hemisphere in pED; while 45 connections were identified as bridges in the right hemisphere and 57 in the left hemisphere in HC.

**Table 3:** The top 15 ranked brain network paths in pED and HC.

**pED**				**HC**			
**Region A**	**Region B**		**Lobe-lobe**	**Region A**	**Region B**		**Lobe-lobe**
CAU	CAU	R-L	S-S	CAU	CAU	R-L	S-S
PUT	CAU	L-L	S-S	THA	HIP	R-R	S-T
CAU	HIP	L-L	S-T	PUT	HIP	L-L	S-T
HIP	PCG	L-L	T-P	MTG	PUT	R-R	T-S
PUT	HIP	L-L	S-T	CAU	ACG	L-L	S-F
CAU	ACG	L-L	S-F	PUT	CAU	R-R	S-S
PCG	PCG	R-L	P-P	PUT	ORBsupmed	L-L	S-F
THA	CAU	L-L	S-S	PCUN	SPG	L-L	P-P
PCUN	MOG	L-R	P-O	PUT	CAU	L-L	S-S
THA	HIP	R-R	S-T	THA	PHG	R-R	S-T
PCG	DCG	L-L	P-F	MOG	CUN	R-L	O-O
THA	CAU	R-L	S-S	DCG	SFGdor	R-R	F-F
MTG	PCUN	R-L	T-P	PCUN	HIP	L-L	P-T
PUT	CAU	R-L	S-S	CAU	IFGtriang	L-L	S-F
CAU	ORBsup	R-R	S-F	THA	CAU	R-R	S-S

### Relationships between degree of amygdala and clinical characteristics

**Figure 6:**
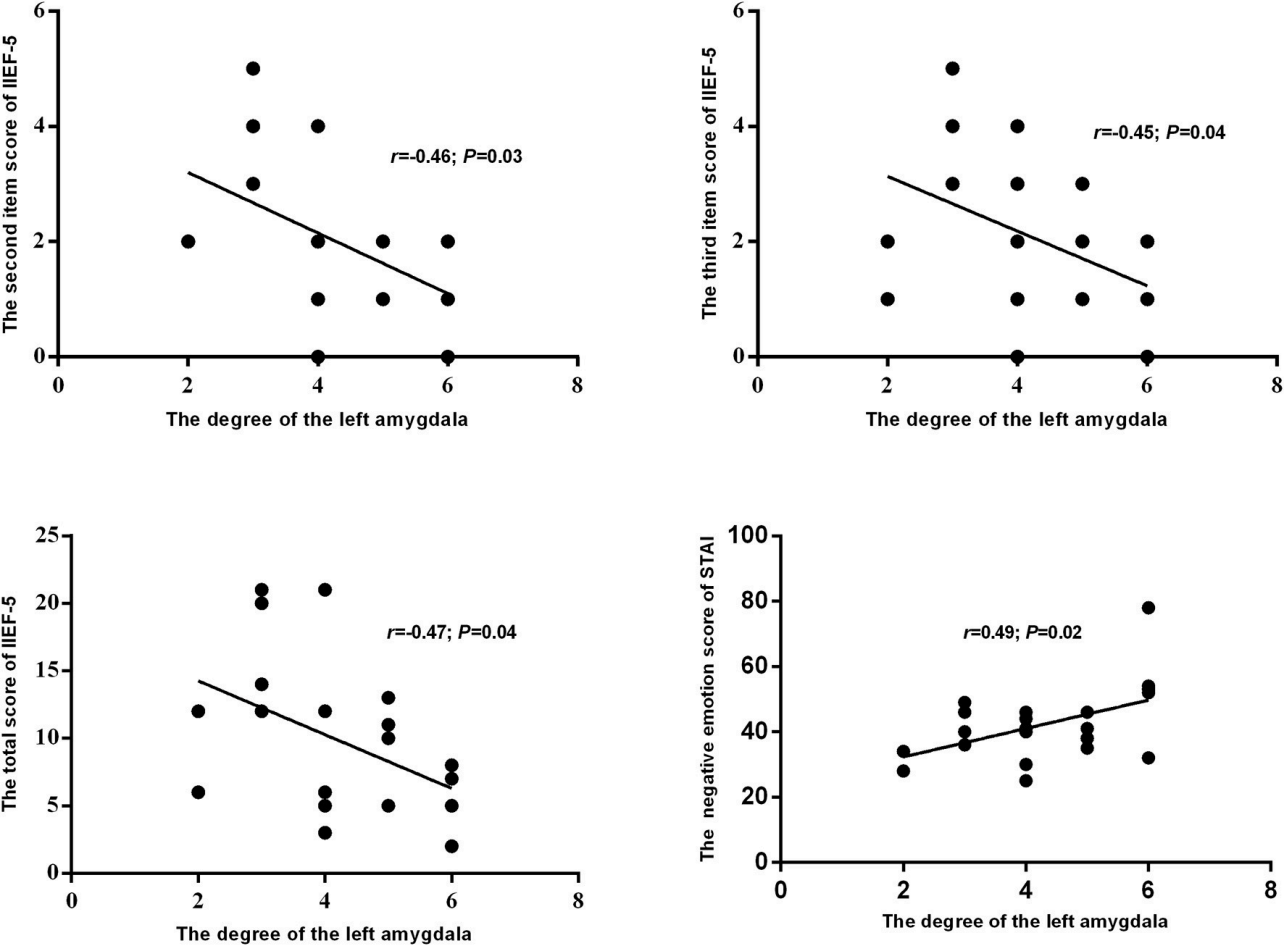
The relationships between the degree of the left amygdala and self-reported emotion and erection.

The results of this study also showed a significantly lower degree of the left amygdala in the networks of pED than in the HC. Furthermore, the degree of the left amygdala of pED showed significantly negative correlations with the total, second and third item scores of IIEF-5, and positive correlation with the negative emotion of STAI (Figure 6).

## Discussion

In the current study, we used RigiScan device to evaluate erectile function of petients and utilized diffusion MRI and graph theoretical approaches to investigate the changes in network properties associated with self-reported erection emotion. The main findings were as follows: (1) all patients showed normal nocturnal erection, however impaired slef-reported erection and negative emotion; (2) impaired connectivity in the left prefrontal-amygdala pathway; (3) lower leftwrd asymmetry in the inferior frontal gyrus; (4) more hub regions and fewer pivotal connections; (5) the degree of the left amygdala was negatively correlated with the self-reported erection and was positively correlated self-reported negative emotion. These findings showed normal nocturnal erection in pED and supported the possibility that the abnormal erection and emotion in pED might be a result of the less efficient connectivity in the prefrontal-amygdala pathway in the left hemisphere.

All patients showed normal nocturnal erection, which indicated the presence of normal neurologic and vascular structures in penis despite the changes in brain connectivity. Therefore, the diagnosis of pED was conducted when the patient was absence of organic ED. However, the abnormal psychological factors, such as negative emotion, may also lead to the development of erectile dysfunction in pED. And the abnormal connectivity in the brain may be related to the effects of the negative emotion on the erectile function of patients with pED. Regretfully, a limitation of our study is that NPT is not performed in healthy control patients with an IIEF score above a certain limit 20.

Previous imaging findings of activation in the prefrontal cortex and amygdala during SA and particularly during penile erection lead researchers to discuss their (the prefrontal cortex and amygdala) involvement especially in emotional component of earlier models of SA (Redouté et al., 2000; Kim et al., 2013). Additionly, the prefrontal-amygdala pathway has been shown to be specifically involved in the emotion regulation in major depressive disorder (Klauser et al., 2015). However, given their fundamental roles in mediating SA and emotion, the interactions between the prefrontal cortex and amygdala relates to erotic emotional processing and the cognitive regulation of SA is hard to disentangle (Walter et al., 2008). The prefrontal cortex playes a vital role in the evaluation of the motivational/emotional information and in the initiation of goal-directed behavior (Barch and Dowd, 2010). Our findings demonstrate that the decreased efficient in the left inferior, superior frontal gyrus, and medial orbital frontal cortex may be involved in the abnormal processing of sexual intensity and the failed control of emotionally driven SA during sexual behaviors. Furthermore, the prefrontal cortex which has been found to be important for sustaining attention (Esterman et al., 2015), is correlated with the cognitive component of the proposed model. The sexual stimulus is categorized as a sexual incentive (Karama et al., 2002), quantitatively evaluated and sustainingly attended through the cognitive network of brain, especially the attentive network. Moreover, the orbitofrontal cortex, is essential during reward processing (Liu et al., 2007), such as the representation of pleasant touch, and activation of the prefrontal cortex may be related to the representation of the pleasant bodily sensations induced by penile tumescence during the onset of penile erection (Francis et al., 1999). Therefore, the decreased connectivity strength between the inferior, superior frontal gyrus, and medial orbital frontal cortex and amygdala may be related to the inhibitory processes of the cognition, including the abnormal appraisal of sexual stimuli (not evaluating stimuli as sexual and desirable) and the decreased attention on sexual stimuli, which preventing the emergence of SA in unsuitable situations.

Previous findings also provide evidences for an important role of the amygdala in regulating human emotion, especially negative emotion and sexual behavior (Cremers et al., 2010; Kim et al., 2013). The neural pathway subserving the recognition of emotion and the processing of erotic visual stimuli, includes associative cortices and limbic system (Kim et al., 2013; Klauser et al., 2015). Indeed, the amygdala receives multimodal sensory input from the prefrontal cortex and the subcortical structures (Likhtik and Paz, 2015). Furthermore, the neurobehavioral model suggestes that the activation of the amygdala is related to the evaluation of emotional content of the complex perceptual information associated with the visual processing of the erotic stimuli (Redouté et al., 2000; Ferretti et al., 2005). Moreover, amygdala is widely believed to be a key structure in mediating interactions of emotional valence and SA (Walter et al., 2008). Therefore, regarding the emotional component of SA, our results also demonstrate that the decreased connectivity strength of amygdala is specifically involved in the abnormal processes of the emotional aspects of penile sensations in pED.

Positive emotions serve important social functions, motivating social engagement and reversing the physiological activation caused by negative emotions (Sturm et al., 2015). Additionally, low levels of positive emotion can give rise to the appearance of some clinical symptoms such as anhedonia, depression, as well as hyposexuality (Gruber et al., 2011). Emotion generating systems including amygdala, initiate rapid emotional responses to positive emotional stimulis, while emotion regulating systems including the prefrontal cortex, promote down-regulation of affective responding (Wager et al., 2008). The left hemisphere of the brain, and the left frontal lobe in particular, play an important role in positive emotion generation (Sturm et al., 2015). Prior studies have found that left hemisphere, especially left frontal injury decrease positive emotion (Sturm et al., 2015). Our results demonstrate that the decreased connectivity strength of the left amygdala is associated with greater self-reported negative emotion, which suggests that left lateralized impaired prefrontal-amygdala pathway is selectively associated with happiness dysregulation. Additionally, our results support that the selective disruption of the prefrontal-amygdala in the left hemisphere, which is widely believed to play an important role in emotion regulation, and may also be specifically involved in mediating SA and inhibited the the development of SA.

## Conclusion

To summarize, our results showed normal nocturnal penile erection and abnormal topological characteristics in pED patients, especially in the left prefrontal-amygdala pathway. In addition, the inferior, superior frontal gyrus, and medial orbital frontal cortex were found to be important for sustaining attention of the cognitive component of the proposed SA model. The amygdala might play an important role in the emotional component of SA model and the decreased self-reported erection and greater self-reported negative emotion might stem from the decreased connectivity strength of the left amygdala. Overall, these findings provide evidences that abnormal integration within the prefrontal-amygdala pathway in the left hemisphere is associated with the impaired erection and negative emotion in pED.

## Author contributions

JC, YC, ZY, QL, and YD designed the experiment; JC, YC, QG, and GC carried out the experiment; JC, ZY, and QL analyzed the experimental results; JC wrote the manuscript.

### Conflict of interest statement

The authors declare that the research was conducted in the absence of any commercial or financial relationships that could be construed as a potential conflict of interest.
