# Supplementary material for: Impaired Prefrontal-Amygdala Pathway, Self-Reported Emotion, and Erection in Psychogenic Erectile Dysfunction Patients With Normal Nocturnal Erection
Source: Front Hum Neurosci. 2018 Apr 24;12:157. doi: 10.3389/fnhum.2018.00157 (PMC5928255; doi:10.3389/fnhum.2018.00157)
Supplement: Supplementary file 1 [file Data_Sheet_1.docx]

**Table 1** Brain regions defined in the Automated Anatomical Labeling template and their corresponding abbreviations used in this study

| Region name | Abbreviation | Region name | Abbreviation |
| --- | --- | --- | --- |
| Precentral gyrus | PreCG | Angular gyrus | ANG |
| Postcentral gyrus | PosCG | Precuneus | PCUN |
| Rolandic operculum | ROL |  |  |
|  |  | Calcarine fissure and surrounding cortex | CAL |
| Superior frontal gyrus, dorsolateral | SFGdor | Cuneus | CUN |
| Superior frontal gyrus, orbital | SFGorb | Lingual gyrus | LING |
| Superior frontal gyrus, medial | SFGmed | Superior occipital gyrus | SOG |
| Superior frontal gyrus, medial orbital | SFGmedorb | Middle occipital gyrus | MOG |
| Middle frontal gyrus | MFG | Inferior occipital gyrus | IOG |
| Middle frontal gyrus, orbital | MFGorb | Fusiform gyrus | FFG |
| Inferior frontal gyrus, opercular | IFGoper |  |  |
| Inferior frontal gyrus, triangular | IFGtri | Anterior cingulate and paracingulate gyri | ACG |
| Inferior frontal gyrus, orbital | IFGorb | Median cingulate and paracingulate gyri | DCG |
| Supplementary motor area | SMA | Posterior cingulate gyrus | PCG |
| Olfactory cortex | OLF | Hippocampus | HIP |
| Gyrus rectus | GRE | Parahippocampal gyrus | PHG |
| Paracentral lobule | PCL | Temporal pole: superior temporal gyrus | TPOstg |
|  |  | Temporal pole: middle temporal gyrus | TPO |
| Heschl gyrus | HES |  |  |
| Superior temporal gyrus | STG | Amygdala | AMY |
| Middle temporal gyrus | MTG | Caudate nucleus | CAU |
| Inferior temporal gyrus | ITG | Lenticular nucleus, putamen | PUT |
|  |  | Lenticular nucleus, pallidum | PAL |
| Superior parietal gyrus | SPG |  |  |
| Inferior parietal, but supramarginal  and angular gyri | IPL | Thalamus | THA |
| Supramarginal gyrus | SMG |  |  |
|  |  | Insula | INS |

**
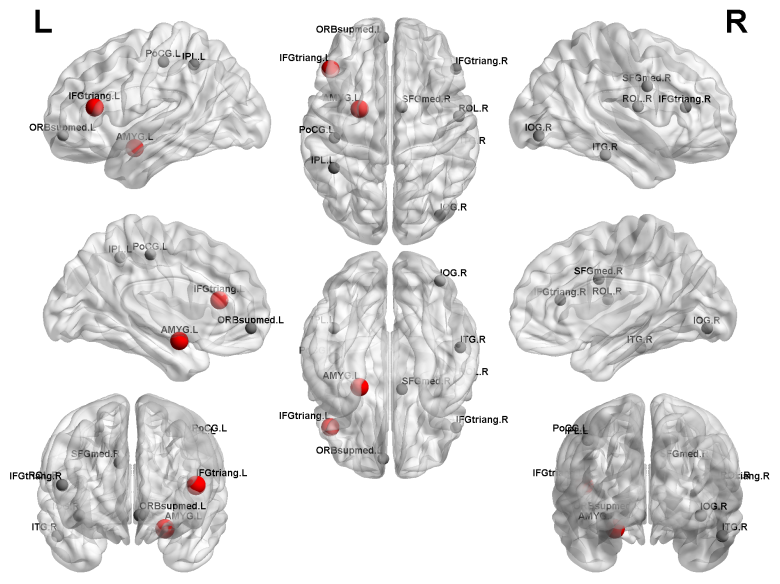
**

**Figure 1** Regions exhibited significant differences in the regional nodal strength. L: left; R: right. The red nodes represented regions that survived FDR correction. The gray nodes represented regions that did not survive FDR correction.

**
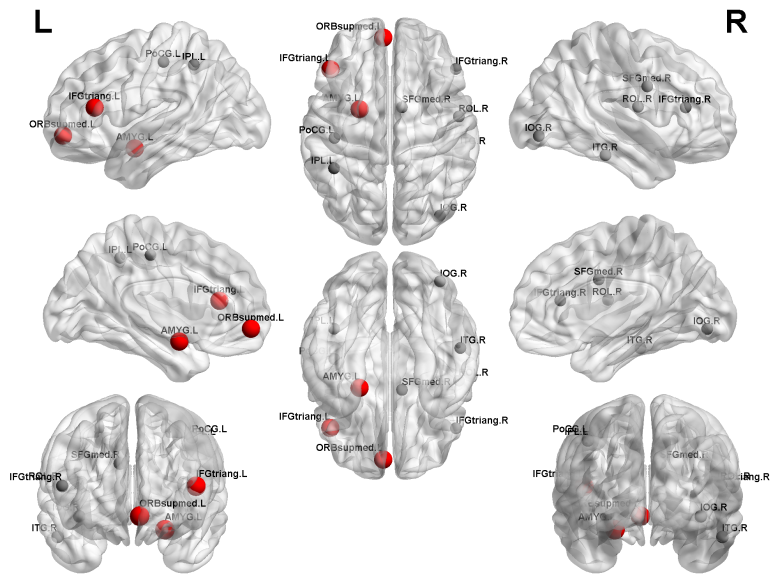
**

**Figure 2** Regions exhibited significant differences in the regional nodal strength. L: left; R: right. The red nodes represented regions that survived FDR correction. The gray nodes represented regions that did not survive FDR correction.
